# Partial Femoral Diaphysectomy With Vastus Lateralis Interposition in a Paraplegic Patient With Severely Debilitating Hip Ankylosis: Low Risks and High Gains?

**DOI:** 10.7759/cureus.35786

**Published:** 2023-03-05

**Authors:** Louis Follet, Lieven Moke, Stijn Ghijselings, Hazem Wafa, Georges Vles

**Affiliations:** 1 Orthopedics, University Hospitals Leuven, Leuven, BEL; 2 Spine Surgery, University Hospitals Leuven, Leuven, BEL; 3 Orthopedic Oncology, University Hospitals Leuven, Leuven, BEL

**Keywords:** diaphysectomy, intercalated resection, hip ankylosis, traumatic heterotopic ossification, neurogenic heterotopic ossification

## Abstract

We present the case of a 56-year-old male unable to sit because of an ankylosed right hip. This ankylosis originated from combined neurogenic heterotopic ossifications (NHO) and traumatic heterotopic ossifications (THO) as a result of a road traffic accident. Because of multiple ossifications, the proximity of neurovascular structures, and chronic pressure ulcers, a resection was deemed unsafe. We opted for a new articulation distal to the ossifications in unstained tissue. A partial femoral diaphysectomy was performed just distal of the lesser trochanter. and the vastus lateralis was rotated in the new articulation. Postoperatively, the patient was able to sit as his hip could flex again.

A partial femoral diaphysectomy with vastus lateralis interposition flap appears to be a valid option in paraplegic patients with extensive heterotopic ossifications (HO) in close proximity to neurovascular structures with a low risk of complications and high gain in hip mobility.

## Introduction

Heterotopic ossification (HO) is the development of lamellar bone at locations where it should not exist. It can be neurogenic heterotopic ossification (NHO) or traumatic heterotopic ossification (THO) in origin or both [1,2]. NHO, caused by spinal cord injury (SCI) or traumatic brain injury (TBI), differs from THO in that the ossifications do not erode the surrounding muscles and neurovascular bundles [1,2]. Although the exact pathophysiology is not completely understood, it is recognized that interleukin 1 (IL-1) overexpression in denervated muscles promotes the formation of NHO [3].

The hip joint is commonly involved with anterior ossifications spreading from the anterior superior iliac spine (ASIS) to the greater and lesser trochanter [2,4]. The ossifications tend to attach to local bony structures and eventually fuse with it, causing hip ankylosis in 3%-8% of patients [1]. The femoral neurovascular bundle is frequently encapsulated by the ossifications, especially in patients with TBI [2].

Patients complain of reduced range of motion, which, if severe enough, can interfere with normal sitting position and balance, cause difficulties with personal hygiene, and even lead to pressure injury. An overall reduction in quality of life (QoL) is generally seen. Noninvasive treatment modalities focus on relieving pressure ulcers, optimizing wound care, and adapting chairs to facilitate sitting. Invasive treatment options include open resection of the ossifications, a (modified) Girdlestone procedure, and even total hip replacement (THR). All are associated with a high risk of complications such as infection, neurovascular injury, and the recurrence of ossifications and pressure sores [5].

This report describes the application of an alternative surgical technique in a paraplegic patient with substantial heterotopic ossifications, resulting in a substantial improvement in their quality of life. To the best of our knowledge, this technique has not been previously reported in the scientific literature. A systematic search in PubMed, utilizing heterotopic ossification, intercalated resection, femur, and partial femoral diaphysectomy as keywords, rendered no relevant results.

## Case presentation

**Figure 1:**
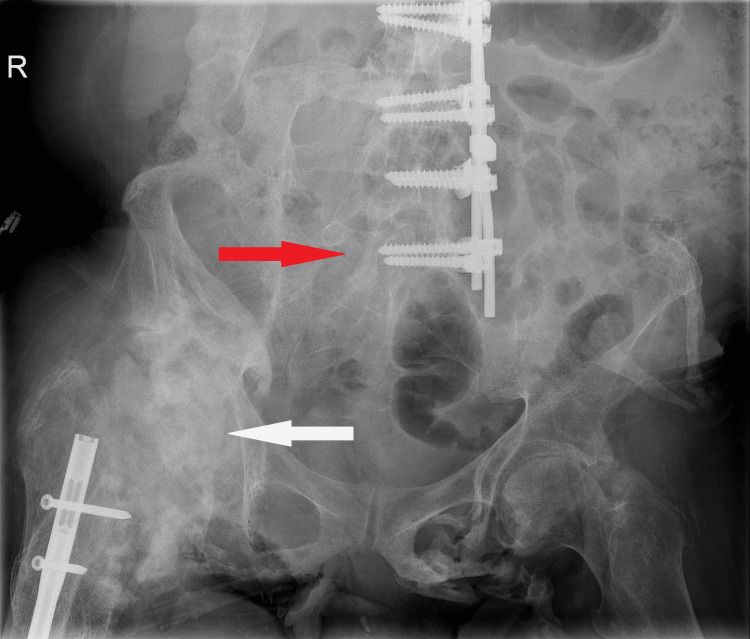
Pelvic radiograph showing the spinal instrumentation (red arrow), the femoral nail and the severe heterotopic ossifications, and the ankylosis of the right hip (white arrow).

A 56-year-old male presented to our clinic because of chronic intractable back pain; a stiff spine with no residual flexion, extension, or rotations; and an ankylosed right hip, leaving him bedridden. Two decades ago, he sustained an unstable burst fracture of T11 and L4 leading to an incomplete spinal cord injury (T9) and a complete spinal cord injury (T10), as a result of a road traffic accident. He underwent a lateral spondylodesis (T10-L5) but remained paraplegic. Eight years later, he underwent intramedullary nailing of a right-sided femoral midshaft fracture after a minor fall. Over the years, as a result of his spinal cord injury and femoral nailing, he started to develop severe heterotopic ossifications, finally resulting in an ankylosed right hip, fixed in 25° flexion (Figure 1).

**Figure 2:**
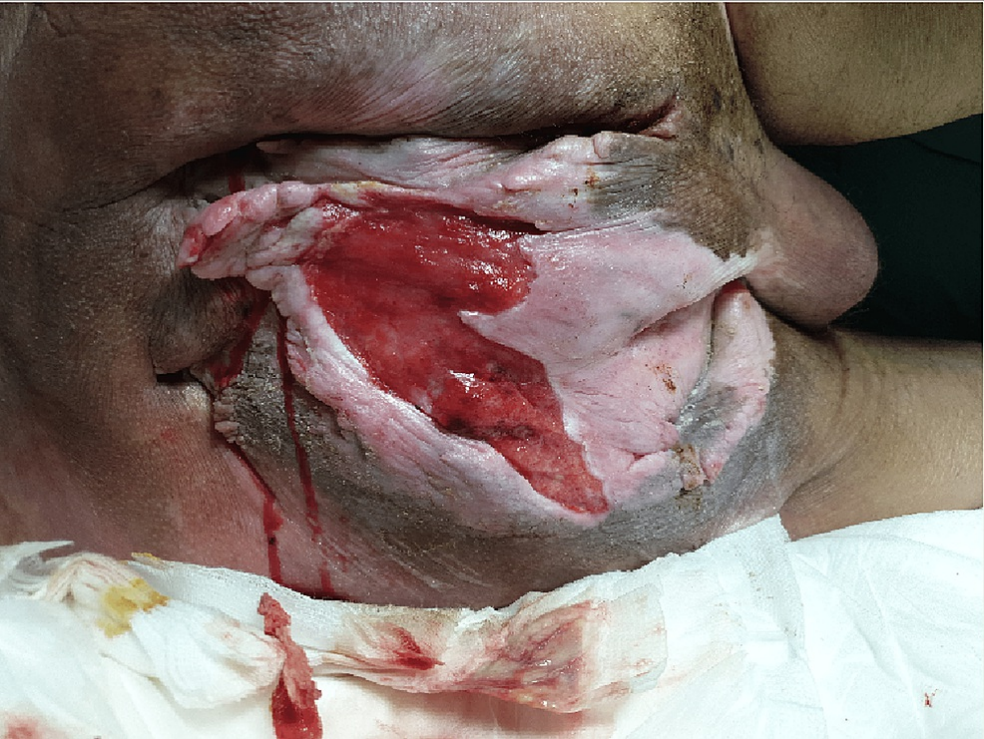
Clinical image of the chronic sacral pressure sore.

Due to his previous spondylodesis and his now fully ankylotic right hip, he was left in a rigid semi-upright position. Any attempt to assume a more flexed position led to severe lumbar pain. This caused him to be bedridden with subsequent deterioration of his chronic sacral pressure sore, which had remained a stable grade 2 pressure injury for the previous 15 years (Figure 2).

**Figure 3:**
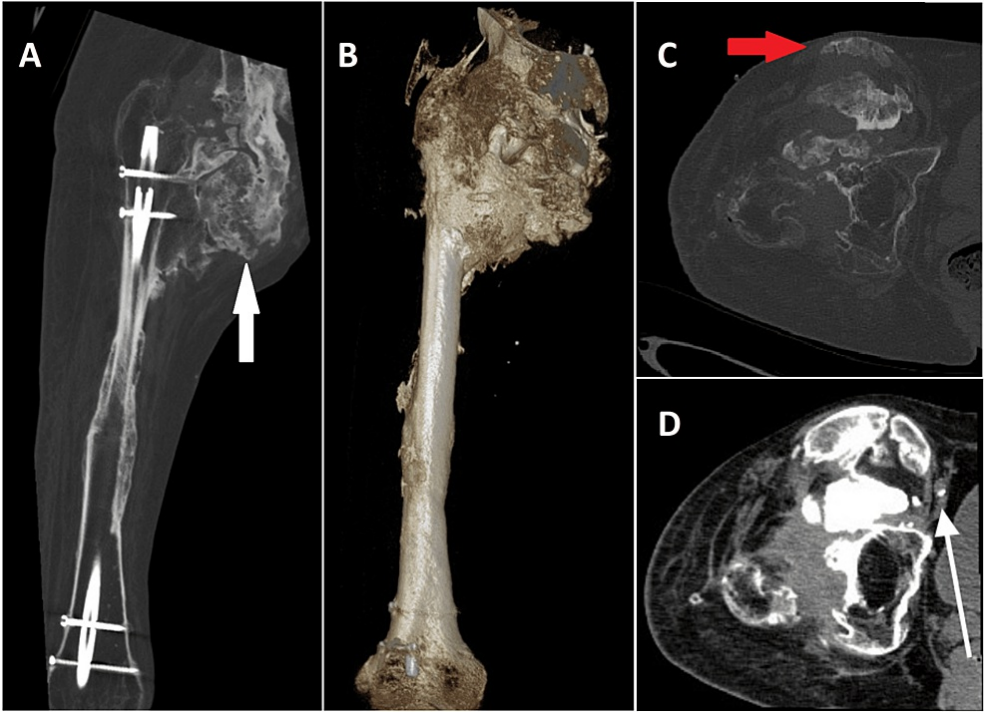
A) Coronal CT of the femur showing the bony bridges (white arrow) between the proximal femur and the right hemi-pelvis, B) 3D reconstruction of the hip ankylosis, C) transversal CT showing the proximity of the anterior calcifications to the skin (red arrow), and D) transversal CT showing the femoral nerve and vessels completely embedded in the NHO (white small arrow). 3D, three-dimensional; NHO, neurogenic heterotopic ossification

On physical examination, the calcifications were visible and palpable just underneath the skin, especially in the groin. A CT scan without contrast showed extensive heterotopic ossifications around the right hip with a number of bony bridges between the proximal femur and the right hemi-pelvis, both anteriorly and posteriorly (Figure 3). It was furthermore revealed that the femoral nerve and blood vessels were enveloped within the heterotopic ossifications. The sacral decubitus wounds were not connected by any fistulae to the hip joint or the spinal instrumentation.

Rationale

This case was discussed at our multidisciplinary team meeting with delegates from hip surgery, spine surgery, trauma surgery, plastic surgery, neurology, rehabilitation medicine, microbiology, and infectious diseases.

A classic Girdlestone procedure would allow the patient to flex his hip again but would come with a very high risk of damaging the neurovascular structures as these were completely embedded in the calcifications. It would also most likely require both an anterior and a posterior approach to the hip given the number of bony bridges between the proximal femur and the right hemi-pelvis. The latter would be nearly impossible given the proximity of the extensive sacral pressure sore. Performing a Girdlestone procedure would furthermore leave an enormous dead space, with just a thin layer of skin remaining anteriorly at the level of the groin. Options for filling this dead space with muscle flaps were discussed; however, the quality of the already severely atrophic vastus lateralis was poor, and using the rectus abdominis would further impede his sitting capacity [6]. A free flap was not an option as he was an avid smoker and not willing to quit. Performing THR was considered to offer no additional benefits and only increase the risk of complications such as dislocation and infection [5].

As our main goal was for his hip to flex between his pelvis and femur again, allowing him to sit in his wheelchair and facilitate the maintenance of hygiene, it was decided to perform a proximal partial femoral diaphysectomy. This would allow us to 1) operate in relatively normal tissue, 2) stay away from the neurovascular structures, and 3) avoid affecting the healing of the extensive sacral pressure sore. In order to prevent the re-union of the bony parts, a vastus lateralis interposition was performed. This proposal was discussed with the patient and his family, and informed consent was obtained.

Surgical technique

**Figure 4:**
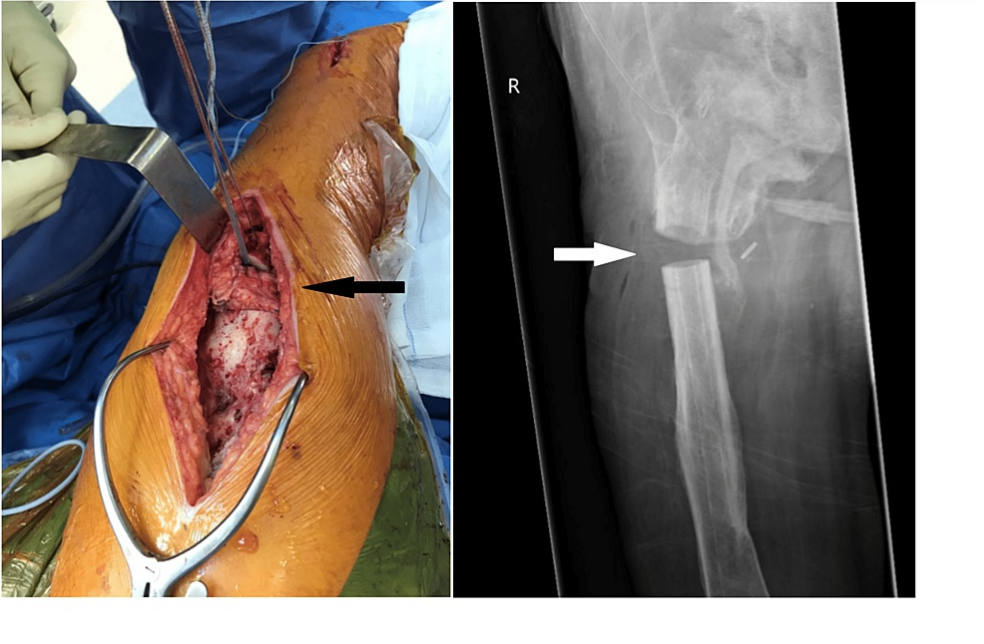
(Left) Intraoperative photograph showing the osteotomy, the vastus lateralis muscle flap interposition, and the transosseous fiber loop to prevent excessive posterior translation of the distal half of the femur (black arrow). (Right) Postoperative radiograph of the femur showing the partial femoral diaphysectomy (white arrow) with good alignment and remaining gap.

Following general anesthesia, 2 g of cefazolin was administered intravenously as infection prophylaxis and continued for 72 hours postoperatively. The patient was positioned in a lateral decubitus position using pelvic supports. After routine prepping and draping, the two distal locking screws were identified using fluoroscopy and removed via the previous stab incisions. Next, a straight lateral approach to the proximal femur was performed. The vastus lateralis was released proximally and mobilized distally. The lateral circumflex femoral vessels were identified and protected while rotating the proximal stump of the vastus lateralis, preserving the blood supply. The two proximal locking screws were identified and removed. The femur was then osteotomized 4 cm distal to the second most proximal screw hole, and a cylinder of the bone with a length of 8 cm was removed. The intramedullary nail was divided using a pneumatic surgical drill (Midas Rex, Medtronic Minimally Invasive Therapies, Minneapolis, MN), and both parts were removed via the osteotomy. Bone wax (B. Braun, Rubi, Barcelona, Spain) was administered to both ends of the osteotomy. The vastus lateralis interposition flap was fixed to the proximal end of the osteotomy by two Healix anchors (Mitek, DePuy Synthes, Raynham, MA) and to the intermuscular septum using the non-resorbable wire Ethibond (Ethicon, Somerville, NJ) (Figure 4). To prevent the posterior translation of the distal half of the femur, a transosseous FiberTape (Arthrex, Naples, FL) loop was sutured to the iliotibial tract. After hemostasis and lavage, the wound was closed in separate layers over a redon, followed by intradermal closure of the skin.

Outcome

**Figure 5:**
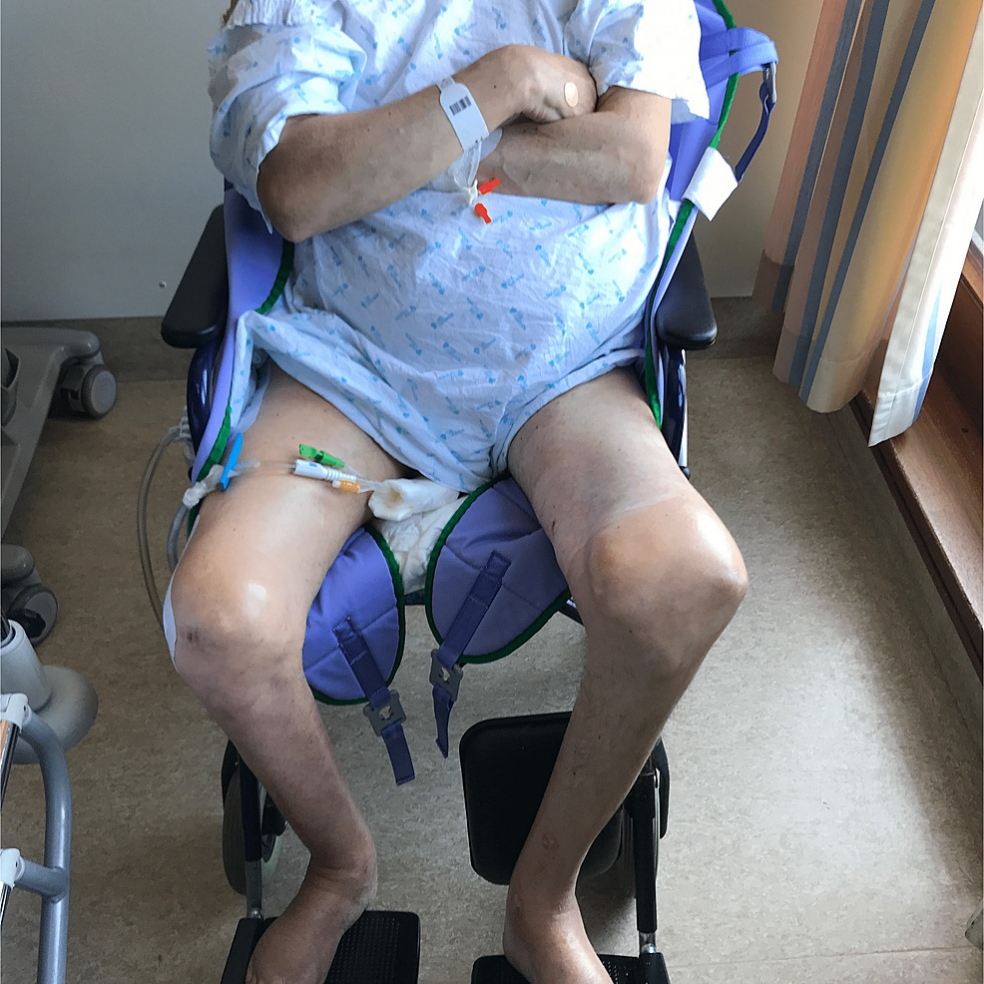
The patient sitting comfortably in his wheelchair, for the first time in years, on the day after the operation.

Immediately after the operation, the patient was able to sit comfortably in his wheelchair again for the first time in years (Figure 5). The amount of time spent out of bed was gradually increased, and over the next two months, from a bed-bound situation, he was able to be on a seated position for two hours in the morning and two hours in the afternoon. His back pain diminished, while his sacral pressure sore improved. His QoL improved dramatically with him now being able to leave the house, do his own cooking, and engage in social activities.

## Discussion

While an open resection is still considered as the golden standard [1,4,7-9], we report on an alternative technique, i.e., partial femoral diaphysectomy with vastus lateralis interposition, to regain hip mobility and sitting ability in a paraplegic patient with severely debilitating hip ankylosis. The procedure was relatively short and straightforward, the postoperative course was uneventful, and the increase in QoL is substantial. It allowed us to reach our goals while minimizing the risk of infection, neurovascular damage, and dead space problems. This is in stark contrast with the open resection where postoperative infections are common at 9%-38% [7].

Despite the good short-term outcome, we had concerns about distalizing the flexion point of the hip, especially the emergence of new bed sores, due to the new articulation. As the proximal part of the femur is fixed at 25° flexion, new articulation is possible at the new pressure point when the patient is seated. The patient’s weight is then divided between the contralateral ischial tuberosity and the pseudo-articulation. These pressure points can be unburdened by a personal adapted wheelchair/seating. A thorough search in the literature did not reveal any studies about the risks of a distalized articulation.

Furthermore, no literature was found about the chances of late fusion of the intercalated resection, which would decrease the mobility of the new articulation. After the resection of the heterotopic calcifications, the recurrence rates are between 3.5% and 57% [1,4,9]. The prevention of necrosis of the vastus lateralis flap is also important. Muscle damage can promote heterotopic ossifications [3]. Therefore, we protected the branches of the lateral circumflex vessels, though there is additional blood supply by the perforating arteries as well.

The possible contraindications for this procedure could be skin or wound problems in the surgical site, which can lead to surgical site infections. Though the surgical technique looks promising, no long-term follow-up is available.

## Conclusions

In conclusion, in paraplegic patients with an ankylosed hip due to heterotopic ossifications in the proximity of neurovascular structures and after comprehensive counseling, partial femoral diaphysectomy with vastus lateralis interposition appears to be a low-risk procedure with high gains. We minimalized the risk of infection, staying clear of the bed sores. We had no dead space to manage, which is not the case after the resection of the heterotopic calcifications. A nice correction of his sitting balance was achieved through the newly regained ability of flexing the hip joint. The fusion of the neo-articulation was prevented by vastus lateralis interposition. We tried to minimize the tissue damage by sparing the nutritional vessels to the vastus lateralis. We should note that this is not a weight-bearing construct, which was not necessary for our paraplegic patient.
